# Role of Interferons in the Development of Diagnostics, Vaccines, and Therapy for Tuberculosis

**DOI:** 10.1155/2017/5212910

**Published:** 2017-06-20

**Authors:** Kai Ling Chin, Fadhilah Zulkipli Anis, Maria E. Sarmiento, Mohd Nor Norazmi, Armando Acosta

**Affiliations:** School of Health Sciences, Health Campus, Universiti Sains Malaysia, 16150 Kubang Kerian, Kelantan, Malaysia

## Abstract

Tuberculosis (TB) is an airborne infection caused by *Mycobacterium tuberculosis* (Mtb). About one-third of the world's population is latently infected with TB and 5–15% of them will develop active TB in their lifetime. It is estimated that each case of active TB may cause 10–20 new infections. Host immune response to Mtb is influenced by interferon- (IFN-) signaling pathways, particularly by type I and type II interferons (IFNs). The latter that consists of IFN-*γ* has been associated with the promotion of Th1 immune response which is associated with protection against TB. Although this aspect remains controversial at present due to the lack of established correlates of protection, currently, there are different prophylactic, diagnostic, and immunotherapeutic approaches in which IFNs play an important role. This review summarizes the main aspects related with the biology of IFNs, mainly associated with TB, as well as presents the main applications of these cytokines related to prophylaxis, diagnosis, and immunotherapy of TB.

## 1. Introduction

### 1.1. Tuberculosis

*Mycobacterium tuberculosis* (Mtb) is a human-restricted pathogen which causes tuberculosis (TB). TB is one of the most common infections worldwide, mostly affecting individuals in low- and middle-income countries [1]. In 2015, 10.4 million cases of TB and more than 1.8 million deaths were reported [1]. About 2 billion people are latently infected with TB worldwide (about one-third of the world's population) and 5–15% of them will develop TB in their lifetime [2]. It is predicted that in the next 20 years, an additional 1 billion people will be infected with TB and 35 million will die unless effective preventive means are provided [3]. TB is an airborne disease transmitted by inhalation of Mtb-containing aerosol droplets from infected secretions of the respiratory airways [4]. Once inhaled, Mtb is phagocytized by alveolar macrophages and has the ability to survive and replicate inside these cells in a modified phagosomal compartment for decades. A strong cell-mediated immune response can effectively inhibit bacterial replication in latently infected individuals [5]. Although the human immune system can control the infection, the prevalence of TB is being sustained by two important factors, that is, (1) human immunodeficiency virus (HIV) infection and (2) the presence of multidrug-resistant (MDR) strains of Mtb [1]. In 2015, about 35% of HIV-infected patients died due to coinfection with TB [1]. These immunocompromised individuals developed active TB due to failure of their immune system to control or eradicate the infection [6]. Additionally, it was estimated that 480,000 people developed multidrug-resistant TB (MDR-TB) in 2015 [1]. It is reported that interferon- (IFN-) mediated innate and adaptive immune responses are involved in the host immune response against TB [7, 8].

### 1.2. Types of Interferons

IFNs are cytokines that carry signals between cells [9]. Generally, IFNs are differentiated according to their molecular structure and classified into three groups depending on the type of receptor through which they signal. Type I IFNs consist of 13 subtypes of IFN-*α*, and single subtypes of IFN-*β*, IFN-*κ*, IFN-*ε*, IFN-*ω*, and IFN-*τ*, which bind to a receptor complex composed of two chains, IFNAR1 and IFNAR2 [10]. IFN-*γ* is the only interferon classified in type II IFN, and it binds to a receptor complex composed of the IFNγR1 and IFN-*γ*R2 subunits [11]. Type III IFNs consist of IFN-*λ*1 (IL-29), IFN-*λ*2 (IL-28A), IFN-*λ*3 (IL-28B), and IFN-*λ*4, which signals through a receptor complex consisting of IL10R2 (also called CRF2-4) and IFNLR1 (also called CRF2-12) [12]. Type I IFNs are expressed upon recognition of bacterial and viral components [13], and type II IFN is induced by IL-12 and IL-2 stimulation [14], while type III IFNs are induced by viral components [15]. This shows that host responses are stimulated not only by pattern recognition receptors but also by cytokine responses [13, 14]. IFNs are released by the host cells to regulate and activate immune response [16]. Type I IFNs are produced by almost every cell in the body (such as leukocytes, fibroblasts, and endothelial cells), while the type II IFN (also known as immune interferon) is produced by T-cells (especially CD4^+^ T-cells) [17]. Type III IFN is produced mainly by epithelial cells such as lung epithelial cells, hepatocytes, and trophoblastic cells [18]. When IFNs are released, they bind to different kinds of receptors which lie on the surface of cells before being drawn into the cytoplasm [19]. This causes a series of intracellular events involving other proteins inside the cell, resulting in the activation of different processes involved in the response to infections [19] (Table 1).

### 1.3. Interferon Downstream Signaling

IFNs activate Janus-activated kinase-signal transducer and activator of transcription (JAK-STAT) signaling pathway to transmit information from extracellular chemical signals to the nucleus resulting in DNA transcription and expression of genes involved in immunity, proliferation, differentiation, and apoptosis [20]. JAKs are intracellular, nonreceptor tyrosine kinases associated with types I, II, and III IFN receptors. When IFNs are released, they bind to specific receptors which lie on the surface of cells, activating JAKs which then autophosphorylate tyrosine residues on the receptors and create binding sites for STATs [20]. Types I and III IFNs activate STAT1 and STAT2, form heterodimers which combine with IFN regulatory factor 9 (IRF9), and form IFN-stimulated gene (ISG) factor 3 (ISGF3) complexes [20–22]. Also, types I, II, and III IFNs can stimulate the formation of STAT1-STAT1 homodimers [20–22]. Both the ISGF3 complexes and the STAT1-STAT1 homodimers are translocated into the nucleus and induce expression of genes via the IFN-stimulated response element (ISRE) or IFN-*γ*-activated site (GAS) promoters, respectively [20–23]. Intracellular signaling pathway for types I, II, and III IFNs are shown in Table 1. In addition to JAK-STAT pathway, type I IFN-activated JAKs can also activate other signaling pathways, such as CRKL-STAT5 complexes, mitogen-activated protein kinase (MAPK) p38, and mediate initiation of mRNA translation via phosphorylation of insulin-receptor substrates (IRS1 and IRS2) [20].

## 2. Role of Interferon in Tuberculosis

### 2.1. Interferon-Mediated Immune Response in TB

Several reports linked the Mtb-enhanced infection to IFN type I-induced effects [8, 24, 25]; in contrast, other reports in mice and humans describe positive effects and inhibition of macrophage alternative activation favoring the protective mechanisms against Mtb infection [26, 27]. Within a few hours postinfection, during the early stage, both types I and II IFNs are produced in similar quantities. This common proinflammatory pathway act synergistically to induce an optimal immune response to TB, in particular, the recruitment, differentiation, and survival of dendritic cells and macrophages in the lungs. These myeloid cells are able to phagocytose and subsequently kill the pathogens acting as the first line of the immune defense system [8]. However, this also indirectly promotes TB infection by providing target cells (especially macrophages) for intracellular growth of Mtb [8]. Mtb is able to evade macrophage responses and develop immune escape mechanisms by inhibiting acidification/maturation of phagosomes and preventing phagosome-lysosome fusion [28]. This enables Mtb to persist inside macrophages, replicate, and spread to new host cells [28]. It is reported that production of type I IFNs (IFN-*α* and IFN-*β*) during TB infection help to promote the disease [24, 25]. They induce the immunosuppressive/macrophage-deactivating cytokine IL-10 and block Th1 immune response and suppress host-protective cytokines such as TNF-*α*, IL-12, and IL-1*β* [24, 25]. Also, when both types I and II IFNs are in similar concentration, type I IFNs limit the expression of IFN-*γ*-induced MHC class II on antigen-presenting cells (APCs) [29].

Several days postinfection, the adaptive immune response to TB is optimally activated, where CD4^+^ and CD8^+^ effector T-cells traffic to the lungs where they produce IFN-*γ* [8]. At this stage, the concentration of IFN-*γ* would be ten times higher compared to that of type I IFNs [8]. Many studies have shown that IFN-*γ* driven Th1 responses are crucial for the immune response in Mtb infection [8]. Since Mtb is a pathogenic intracellular microorganism, Th1 type cytokines play a major role in stimulating cell-mediated immune responses for the development of host protection. Under these conditions, IFN-*γ* becomes the predominant immunomodulatory regulator by recruitment of T-cells, induction of expression of MHC class II molecules, augmentation of APCs, and control of Mtb growth [8]. In addition, IFN-*γ* promotes cellular proliferation, cell adhesion, apoptosis, and autophagy [30]. IFN-*γ* increases mycobactericidal activity in the infected macrophages by inducing respiratory burst with production of reactive nitrogen intermediates (RNI) and reactive oxygen intermediates (ROI) [31]. Type III IFNs are not essential for Mtb infection control, but may contribute to the modulation of Th1/Th2 immune responses to this pathogen [25]. The concentration of type III IFNs had been reported to be increased in the sputum of pulmonary TB patients compared to that of latently infected and uninfected healthy individuals, which suggest the possibility of the production of this cytokine by inflammatory cells under the influence of Mtb products [32]. The balance between IFN-*γ* and other cytokines, such as IL-10 and other Th2 cell cytokines, is likely to influence the disease outcome [8]. Overall, IFNs seems to be important modulators of host immune responses for protection against TB, and thus have been used in diagnostic, therapeutic, and vaccination approaches. In the following sections, we will review the different applications related to IFNs on these three categories (Table 1).

### 2.2. Use of Interferons in Diagnosis

After infection with Mtb, the bacteria are contained by the host immune system and persist in subclinical status with minimal replication and no clinical manifestations of the disease [33]. In this dormant stage, also referred to as latent TB, the bacteria can persist for decades [33]. In situations where the individual's immunologic status is compromised, Mtb may begin to replicate, resulting in the reactivation of TB [34]. At this stage, the gold standard for diagnosis of active TB is the bacteriologically confirmatory test using sputum as biological sample [35]. However, direct acid-fast microscopy using Ziehl-Neelsen staining has low sensitivity (requires approximately 5000–10,000 bacilli per 1 mL sputum for detection), and culture is laborious and time consuming, taking between 2 and 8 weeks to give a positively result [36]. Alternatively, a faster way of identification can be achieved using nucleic acid amplification tests (NAATs) [37]. Both tests require sputum which is not always available (especially in infants and young children) [38].

The latent TB diagnosis is of paramount importance from the epidemiological point of view as it allows the treatment to prevent the risk of future development of active TB by 60–90%. Such treatment could avoid the estimated 20–30 new infections that are produced from each active TB case [39].

Currently, only two diagnostic methods are used to diagnose latent TB, that is, tuberculin skin test (TST) and IFN-*γ* release assays (IGRAs) [40]. TST (also known as the Mantoux test) was developed more than 100 years ago. Purified protein derivative (PPD), a crude mixture of the culture filtrate of Mtb, is used for intradermal injection on the forearm of the individual, followed by the measurement of the induration produced at the site of injection after 48 to 72 hours [41]. The results are expressed as the size of the induration in the site of injection after this period [40]. Depending on the risk group of the individual, different cut-off values for positivity are established [40]. False-positive results are reported by this test due to the presence in the PPD preparation, of nonspecific antigens, which generate false-positive results associated with previous BCG vaccination, and contact with environmental mycobacteria [42]. Another drawback of this test is the high rate of false-negative results in immunosuppressed individuals [42].

Numerous studies have shown that one of the important hallmarks of the immune response to Mtb infection is the release of IFN-*γ* by T-cells [8]. Based on the presence of significant IFN-*γ* responses upon Mtb infection, the evaluation of the response of immune cells to specific Mtb antigens has been used as an indicator of TB infection in diagnostic methods known as the IFN-*γ* release assays (IGRAs) [43, 44]. There are two types of commercial IGRAs available, that is, (1) T-SPOT.TB assay (Oxford Immunotec, UK), an enzyme-linked immunosorbent spot (ELISPOT) test that uses peripheral blood mononuclear cells and (2) QuantiFERON-TB Gold In-Tube assay (Cellestis Ltd., Australia), an enzyme-linked immunosorbent assay (ELISA) that uses whole blood [43, 44]. Both assays use specific stimulating antigens from Mtb, that is, culture filtrate protein 10 (CFP-10) and early secretory antigenic target 6 (ESAT-6), with an additional antigen, TB7.7, included in the QuantiFERON-TB test [45]. When sensitized, memory/effector T-cells from a blood sample, incubated with these proteins, are stimulated to produce IFN-*γ*, and the results are measured after 8 hours (T-SPOT.TB) or 16 hours (QuantiFERON-TB Gold) [45]. T-SPOT.TB is more sensitive (92.0–94.1%) than QuantiFERON-TB (83.0–89.0%) in detecting pulmonary TB patients, and the latter has a poor sensitivity in individuals more than 60 years old [46, 47]. Even though the assays are specific for Mtb and are not influenced by previous BCG vaccination, cross-reactivity have been observed towards some non-TB strains, such as *M. flavescens*, *M. kansasii*, *M. szulgai*, and *M. marinum* [48]. False-negative results may occur in HIV-infected and immunosuppressed patients as they are unable to mount a satisfactory T-cell response, and their production of IFN-*γ* is low [49]. Even though, IGRAs are reported to have higher sensitivity and specificity compared to TST [50, 51]. IGRAs is not fully suitable for children under the age of five as insufficient IFN-*γ* is produced at this age group [52]. However, a recent study reported IFN-*γ* production in young children resulting in slightly more sensitivity of IGRAs compared to that of TST [53]. Although both TST and IGRAs cannot differentiate between latent and active TB, differential diagnosis can be done through clinical and radiologic evaluation [54]. Despite the advantages of using IGRAs as diagnostic tests for latent TB, their future prospects remain uncertain in low- and middle-income countries since they have comparable performance with TST but with higher cost and complexity [55].

### 2.3. Use of Interferons in Therapy

In low-resource settings, inconsistent drug supply and weak TB-control infrastructure can lead to the generation of TB-drug resistance [56]. Once an Mtb strain develops resistance to first-line antibiotics (at least isoniazid, rifampicin), it is defined as MDR-TB [56, 57]. XDR-TB involves MDR-TB, with resistance to any fluoroquinolones and at least one of the injectable second-line drugs [56, 57]. The second-line treatments are less potent and less tolerable compared to first-line treatments, and the usage of these drugs is associated with adverse side-effects and the possibility of lung resection surgery [56].

As alternative treatment of drug resistance TB, therapeutic approaches using IFN-*γ* have been reported [58]. IFN-*γ* is a heterogeneous glycoprotein with molecular weight ranging from 34 to 50 kDa [59]. As stated previously, IFN-*γ* is an important modulator for Th1 immune responses and probably plays an important role in conferring protection against Mtb [8]. Therefore, it is conceivable that recombinant IFN-*γ* could be used for treatment of TB [58]. Condos et al. [58] conducted a study by treating pulmonary TB patients who did not respond to their treatment with IFN-*γ* via aerosol. Using aerosol administration, IFN-*γ* can safely be delivered to the lower respiratory tract without systemic side effects [58]. The treatment helped to decrease the bacterial burden in the lungs, where the patients' sputum smears became negative during the 4-week intervention, and some patients even showed diminished cavitary lesions (suggesting that IFN-*γ* has antifibrotic effect) [58]. However, the effect of exogenous IFN-*γ* was short lived as sputum smears became positive after 1 to 5 months upon discontinuation of treatment [58]. These results highlighted the need to study IFN-*γ* as long-term therapy while evaluating its adverse side effects, tolerability, and therapeutic effects [58]. According to Gao et al. [60], even after 6 months of IFN-*γ* treatment to prevent relapse, no substantial side effects were observed, and adjunctive therapy with IFN-*γ* by aerosol help to improve chest radiographic alterations, in contrast to IFN-*γ* administered intramuscularly or subcutaneously. One report however demonstrated positive outcome of intramuscular administration of IFN-*γ* in pulmonary MDR-TB although definitive conclusions could not be drawn due to the small number of patients studied [61]. Another study demonstrated no significant improvements in clinical, radiographic, microbiologic, or immunologic parameters when IFN-*γ* was administered subcutaneously in chronic and advanced MDR-TB patients [62]. In contrast, a patient with acute lymphocytic leukemia, who had refractory brain MDR-TB and did not respond to anti-TB drugs and steroid treatment for 11 months, showed improvement in brain and chest radiographic alterations after 5 months of adjunctive therapy with IFN-*γ* administered subcutaneously; and complete resolution of the lesions in the brain and spinal cord were obtained after 12 months of therapy [63]. Overall, these studies showed that adjunctive therapy with IFN-*γ* by aerosol, intramuscular, or subcutaneous routes could be useful to treat MDR-TB patients, but further controlled clinical trials are needed in order to establish the role of IFN-*γ* in the treatment of TB [58, 60–63].

Other studies have reported that adjunctive therapy using IFN-*α* was useful in the treatment of TB patients [64, 65]. IFN-*α* combined with antimycobacterial therapy showed favorable results in treating pulmonary TB via aerosol administration [64, 65] and in diabetic MDR-TB patients via intramuscular injection [66]. This is probably because IFN-*α* helps to induce Th1 polarization in responding T-cells and increased production of IFN-*γ* [26]. Also, type I IFNs can confer protection against Mtb infection in mice in the absence of IFN-*γ* signaling by inhibiting alternative macrophage activation, which, when present, may increase the host susceptibility to TB [27]. Although low concentrations of type I IFNs seem to be required during the early stages of bacterial infection to initiate adaptive immune responses, high concentration of type I IFNs may induce Th2 immune responses, enhance production of immunosuppressive molecules, and reduce responsiveness of macrophages to activation by IFN-*γ* [67]. This suggest that the concentration of type I IFNs administered should be monitored if incorporated in a therapeutic schedule. Previous studies have also shown that PEGylated-IFN-*α* therapy, which is the first-line choice of treatment for chronic hepatitis B, C, and D, induces weight loss and anorexia and indirectly increases the risk of TB reactivation, resulting in severe pulmonary TB [68–70]. Thus, individuals who come from high-risk countries should be tested for immunodeficiency (depletion of CD4^+^ T-cells) or latent TB prior to IFN-*α* therapy to prevent TB reactivation.

### 2.4. Use of Interferons in Vaccines

The use of IFNs in TB vaccine development has been dominated by IFN-*γ* with very little reports directly related to other IFNs.

#### 2.4.1. IFN-*α*

The importance of the combined influence of IFN-*α* and IFN-*γ* on human neonatal monocyte-derived dendritic cells to induce the production of IL-12 as an important element for the shift towards a CD4+ T-cell Th1 polarization has been advocated as one of the main effects of BCG vaccination [71].

In an application not directly related to TB vaccine development, BCG expressing IFN-*α*-2b has been developed as an experimental immunotherapeutic alternative to BCG for bladder cancer. BCG secreting IFN-*α*-2b induced higher levels of IFN-*γ*, TNF-*α*, IL-12, and lymphoproliferation as well as increased antiproliferative and cytotoxic effect on bladder cancer cells in vitro [72].

#### 2.4.2. IFN-*γ*

The two main applications of IFNs on TB vaccine development are (1) the use of IFN-*γ* directly as adjuvant and (2) the use of vaccine candidates designed to induce the production of this cytokine after immunization.

*(1) IFN-γ as Adjuvant*. Administration of an optimal IFN-*γ* dose has been shown to enhance Th1-type immunity induced by Ribi adjuvant, resulting in an improved response against a cocktail of several Mtb antigens. However, the adjuvant effect of IFN-*γ* was dose dependent. A dose of 5 *μ*g of IFN-*γ* per mouse per immunization gave optimal protection, whereas lower or higher amounts (0.5 or 50 *μ*g/mouse) of IFN-*γ* failed to enhance protection [73]. In another approach, using recombinant BCG coexpressing Ag85B, ESAT-6, and mouse-IFN-*γ*, an effective protection against Mtb was achieved in C57BL/6 mice [74].

*(2) Vaccine-Induced IFN-γ*. Vaccine candidates based on different technological platforms have been designed to elicit the production of IFN-*γ* after immunization; among the most relevant strategies is the use of IFN-*γ* inducing recombinant cytokines as adjuvant, fusion proteins, genetic constructions with cytokines, or live vectors expressing cytokines related to the induction of IFN-*γ*.

The use of IL-12 and other cytokines for the induction of Th1 immune responses via induction of IFN-*γ* have also been attempted in experimental studies of TB vaccine development [75]. The combination of IL-12 with BCG vaccination induced an increase in IFN-*γ* production and protection compared to BCG in challenge experiments with Mtb in mice [76]. Similarly, DNA immunization protocols incorporating the IL-12 gene either separately or fused with different Mtb antigens and combined with BCG vaccination induced significant levels of IFN-*γ* and protection against Mtb in challenge experiments in mice [77–80]. Likewise, recombinant *M. smegmatis* and BCG expressing Mtb antigens and IL-12 induced increased production of IFN-*γ* and protection against Mtb in mice [81, 82].

IL-15 is another cytokine implicated in the induction of IFN-*γ* production which had been used in experimental vaccines against TB [83]. The immunization with recombinant BCG secreting a fusion protein composed of IL-15 and Ag85B in mice was associated with IFN-*γ* production and protection upon challenge with Mtb [84]. In another approach, the combination of five Mtb antigens with IL-15 expressed in Modified Vaccinia Ankara in different prime-boost schedules induced the production of IFN-*γ* and protection against Mtb in mice [85].

IL-21 has been reported as an important element in the protection against TB and is associated with the induction of IFN-*γ* production [86]. DNA vaccination inducing the expression of fusion proteins containing Mtb antigens and IL-21 enhanced the production of IFN-*γ* and protection against TB in mice in a prime-boost schedule with BCG [87, 88].

IL-23 is another cytokine involved in the induction of IFN-*γ* that has been used in the development of experimental vaccines against TB [89, 90]. Plasmids containing the IL-23 gene administered with DNA vaccines including Mtb antigens increase the production of IFN-*γ* and protection against TB induced by the immunization with such vaccines [90].

Autophagy is a mechanism of great importance in the defense against TB [91–93], which supported the implementation of strategies to exploit this phenomenon in TB vaccine development [94–96]. Lactic acid bacteria together with Mtb antigens increased the autophagy of human mononuclear phagocytes by increasing IFN-*γ* and nitric oxide (NO) levels together with the inhibition of Th2 cytokines involved in the blockage of autophagy [94]. DNA vaccines incorporating autophagy inducers increase the production of IFN-*γ* and the protection against TB in mice [95, 96].

The induction of apoptosis is another strategy explored for the control of Mtb infections, and, in fact, the evasion of apoptosis is suggested to be one of the main strategies for Mtb to escape the immune response [97–100]. Hence, several approaches to TB vaccine development employing the use of proapoptotic vaccine candidates in the form of DNA vaccine, recombinant Mtb, or recombinant BCG platforms have been tested, demonstrating the induction of IFN-*γ* production and protection against Mtb in mice [101–104].

Other strategies directed to improve the Th1 immune responses in TB vaccine development had been attempted. IgG immunocomplexes containing Mtb antigens or IgG Fc fusions with multistage antigens from Mtb, aimed to increase the interaction with APCs, have been tested in mice which induced protection associated with increase of IFN-*γ* production [105, 106].

Although numerous studies have suggested the positive association between the induction of IFN-*γ* responses and protection against TB, several studies have failed to demonstrate such a correlation. Some studies even demonstrated detrimental effects of IFN-*γ* on protection [107–113]. These contradictory findings highlight the lack of a reliable correlate of protection for TB, which reflects the current efforts to determine more robust and consistent correlates of protection for this disease [108].

## 3. Conclusions

Interferons, especially IFN-*γ*, are important immunomodulators in the pathogenesis of Mtb. It helps to activate macrophages and promote a range of cell-mediated immune mechanisms. IFN-*γ* production in infected individuals has been used, as a key element in IGRAs, and represents a valuable tool for the specific detection of latent TB. IFN-*γ* has also been used as adjuvant therapy in TB patients when conventional therapy failed. Besides IFN-*γ*, another cytokine, IFN-*α*, an important signaling protein to recruit myeloid cells during innate immunity, has also been used as adjunctive therapy in TB, but its potential for treatment remains uncertain. In the TB vaccine area, the use of IFN-*γ* as adjuvant or strategies that induce the production this cytokine has been the most popular approach used to induce Th1 polarization and protection, although the role of IFN-*γ* as TB correlate of protection is still debatable.

## Figures and Tables

**Table 1:** Comparison of type I, type II, and type III interferons.

		Type I IFN	Type II IFN	Type III IFN
Source of stimulation		Bacterial and viral components [13]	IL-12 and IL-2 [14]	Viral components [15]
Source of production		Every cell in the body (leukocytes, fibroblasts, and endothelial cells) [17]	T-cells (especially CD4^+^ T-cells) [17]	Epithelial cells [18]
Type		IFN-*α*, IFN-*β*, IFN-*κ*, IFN-*ε*, IFN-*ω*, and IFN-*τ* [10]	Only IFN-*γ* [11]	IFN-*λ*1 (IL-29), IFN-*λ*2 (IL-28A), IFN-*λ*3 (IL-28B), and IFN-*λ*4 [12]
Receptor		IFNAR1 and IFNAR2 [10]	IFN*γ*R1 and IFN-*γ*R2 [11]	IL10R2 (also called CRF2-4) and IFNLR1 (also called CRF2-12) [12]
Intracellular signaling	JAK	JAK1, TYK2 [20]	JAK1, JAK2 [20]	JAK1, TYK2 [21]
	STAT	STAT1, STAT2 [20]	STAT1 [20]	STAT1, STAT2 [21]
	Translocation complex to nucleus	(i) IFN-stimulated gene factor 3 (ISGF3) [20] (ii) STAT1-STAT1 homodimers [20]	STAT1-STAT1 homodimers [20]	(i) IFN-stimulated gene factor 3 (ISGF3) [22] (ii) STAT1-STAT1 homodimers [22]
	Promoters stimulated	(i) IFN-stimulated response element (ISRE) [20] (ii) IFN-*γ*-activated site (GAS) [20, 23]	IFN-*γ*-activated site (GAS) [20]	(i) IFN-stimulated response element (ISRE) [22] (ii) IFN-*γ*-activated site (GAS) [22]
Function in tuberculosis		(i) Induce the immunosuppressive/macrophage-deactivating cytokine, IL-10 [24, 25] (ii) Either block [24, 25] or polarize [26] Th1 immune response (iii) Suppress host-protective cytokines such as TNF-*α*, IL-12, and IL-1*β* [24, 25] (iv) Limit the expression of IFN-*γ*-induced MHC class II on APCs [29] (v) Synergistic effect with IFN type II promoting protection against Mtb infection in mice [8] (vi) Inhibition of alternative macrophage activation [27]	(i) Stimulate Th1 type cytokines [8] (ii) Recruitment of T-cells [8] (iii) Induction of expression of MHC class II molecules and augmentation of APCs [8] (iv) Promotes cellular proliferation, cell adhesion, apoptosis, and autophagy [30]	Not essential for Mtb infection control, but may contribute to the modulation of Th1/Th2 immune responses [25]
Use in diagnosis		No report	IFN-*γ* release assays (IGRAs) [43–55]	No report
Use in therapeutics		Adjunctive therapy with IFN-*α* by aerosol route to treat pulmonary TB. Precaution need to be taken while treating immunodeficiency patients as it may lead to TB reactivation [26, 27, 64–70]	Adjunctive therapy with IFN-*γ* by aerosol or subcutaneous routes to treat pulmonary TB or extrapulmonary TB [58–63]	No report
Use in vaccine		Combination of IFN-*α* and IFN-*γ* enhance production of IL-12 which induce CD4^+^ T-cell Th1 polarization [71]	(i) Use as adjuvant to induce Th1 immunity [73, 74] (ii) Development of fusion proteins, genetic constructions, or live vectors expressing cytokines related to the induction of IFN-*γ* [75–113]	No report
